# Supplementary figures and images for: Inter-ethnic genetic variations and novel variant identification in the partial sequences of CYP2B6 gene in Pakistani population
Source: PeerJ. 2021 Jul 23;9:e11149. doi: 10.7717/peerj.11149 (PMC8312491; doi:10.7717/peerj.11149)

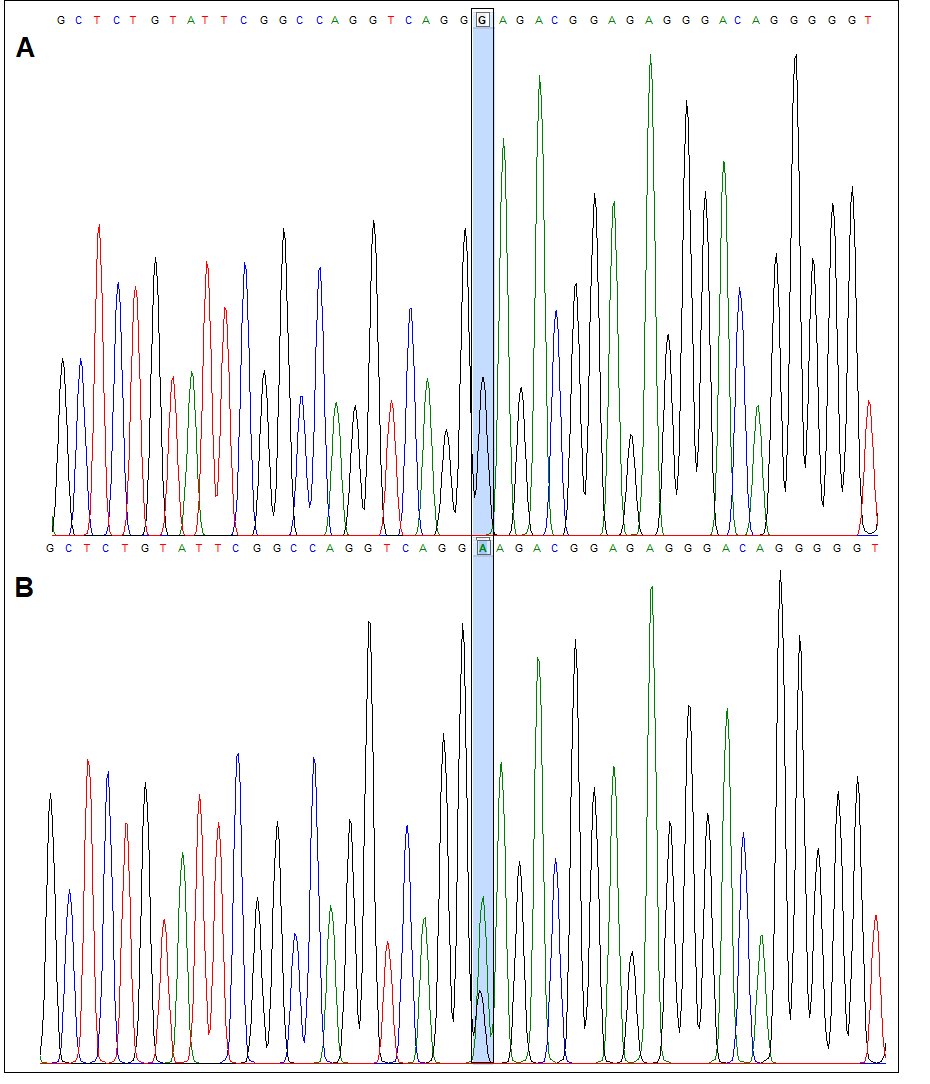

Supplement: Supplemental Information 2 — Chromatogram of nucleotides sequence: (A) Wild type (B) Heterozygous novel SNP (Chr19: 41007072 G→A) detected in a sample from Balochi ethnic group. [file peerj-09-11149-s002.png]

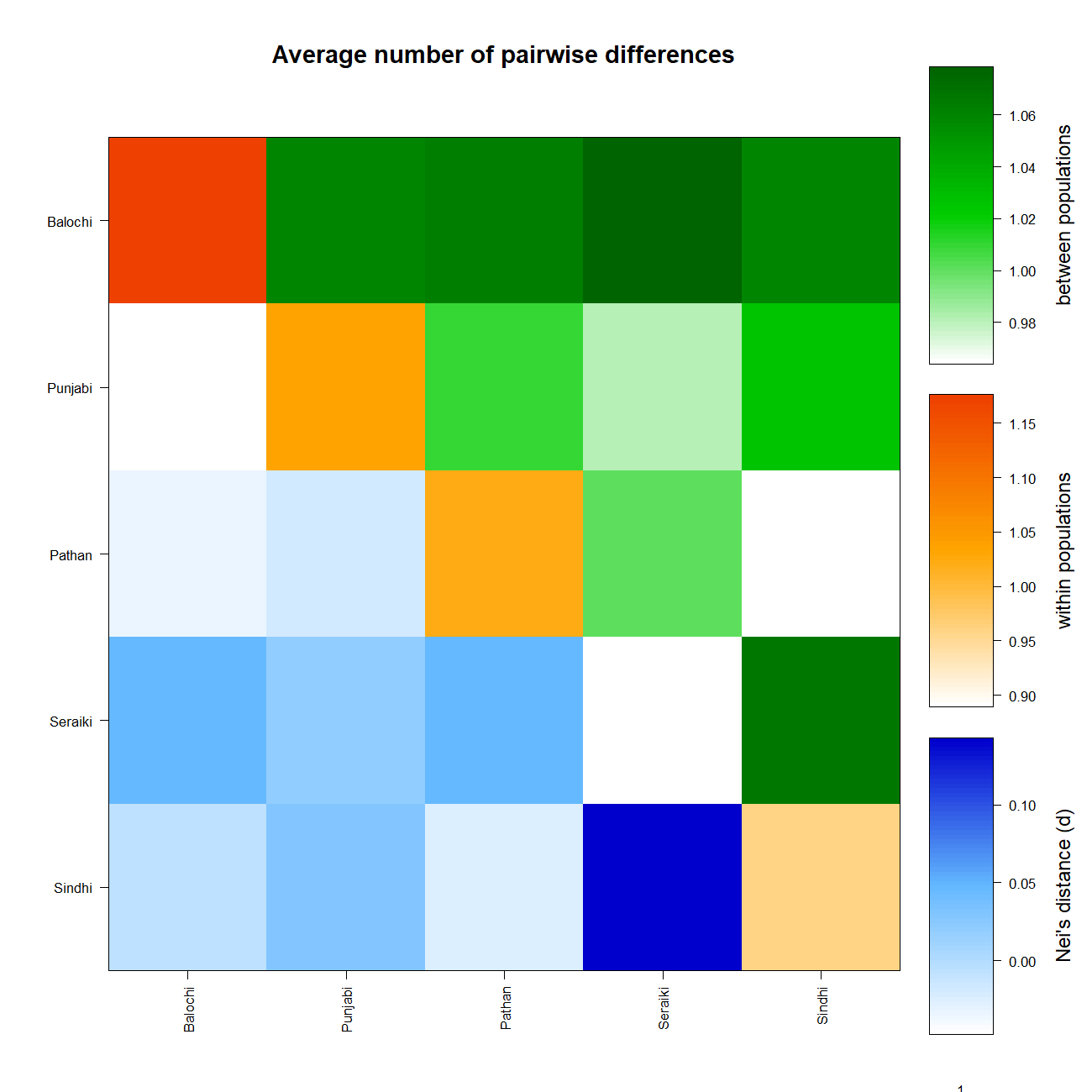

Supplement: Supplemental Information 3 — Pairwise differences (πxy) among the Pakistani ethnic groups—Between sampled population groups (Green above diagonal); within-population πxx (orange diagonal) and the net number of nucleotide differences among population’s groups (Nei distance DA) (blue below diagonal). [file peerj-09-11149-s003.png]

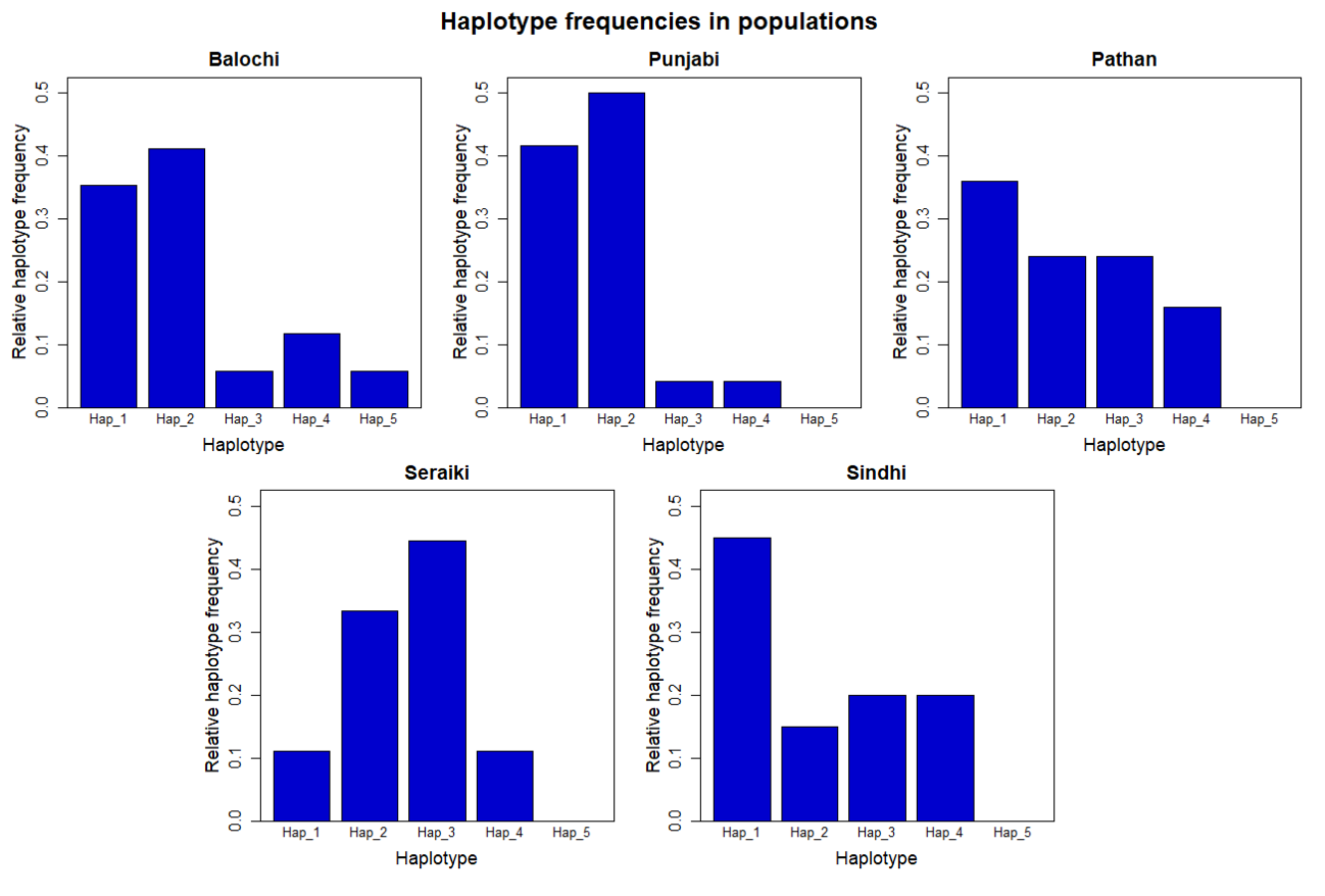

Supplement: Supplemental Information 4 — Detected haplotype frequencies in five ethnic groups samples from the Pakistani population. [file peerj-09-11149-s004.png]

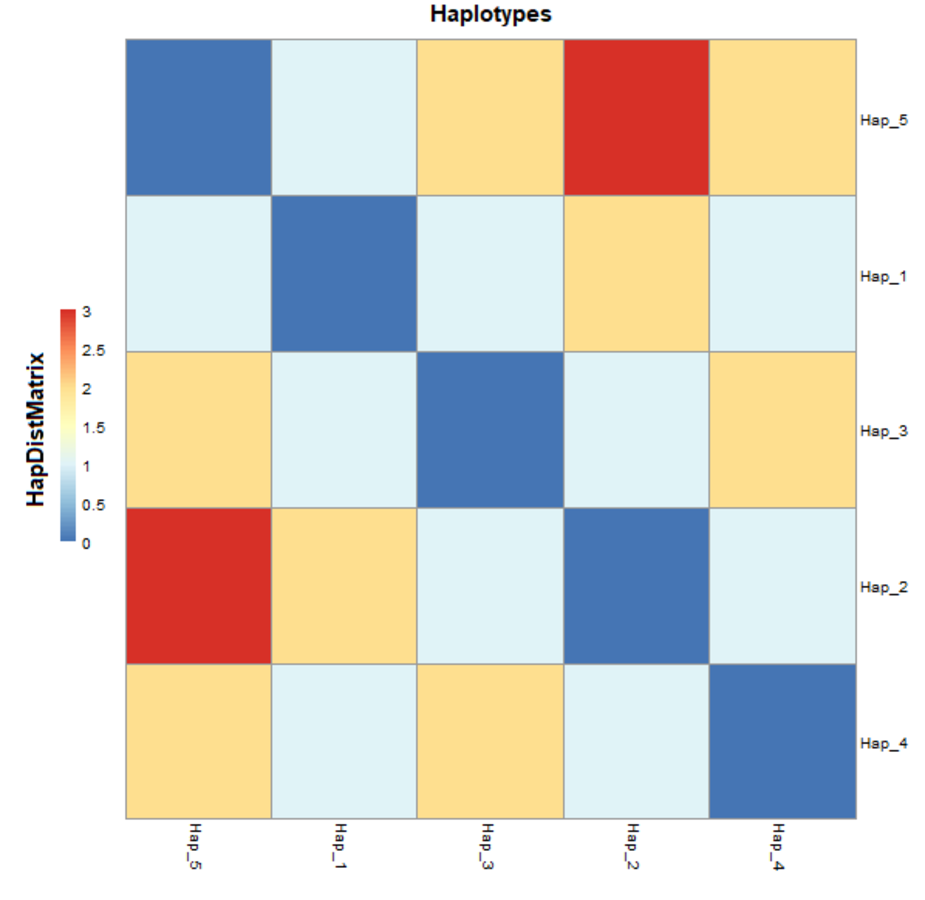

Supplement: Supplemental Information 5 — HapDistMatrix: Depicted pairwise difference among haplotypes of five ethnic groups of Pakistani population. [file peerj-09-11149-s005.png]
